# Use of ecological momentary assessment via wearable devices for detecting acute suicide risk in psychiatric inpatients

**DOI:** 10.3389/fpsyt.2026.1849140

**Published:** 2026-07-01

**Authors:** Yourack Lee, ByeongChang Jeong, Cheol E. Han, Hyun-Ghang Jeong

**Affiliations:** 1 Medical Device Usability Test Center, Korea University Guro Hospital, Seoul, Republic of Korea; 2 Department of Electronics and Information Engineering, Korea University, Sejong, Republic of Korea; 3 CyberBrain Research Section, Electronics and Telecommunications Research Institute, Daejeon, Republic of Korea; 4 Division of Smart Energy Convergence Engineering, Korea University, Sejong, Republic of Korea; 5 Department of Psychiatry, Korea University Guro Hospital, Korea University College of Medicine, Seoul, Republic of Korea

**Keywords:** digital phenotyping, ecological momentary assessment, machine learning, suicide, wearable device

## Abstract

**Background:**

Individuals with acute psychiatric disorders attempt suicide or engage in self-harm even during inpatient hospitalization. Despite ongoing clinical monitoring, accurately identifying fluctuating suicide risk remains challenging in acute psychiatric inpatient settings. Recently, ecological momentary assessment (EMA) has been increasingly investigated as a promising approach for predicting suicide risk by capturing dynamic changes in patients’ mental states. This study investigated whether wearable-derived passive EMA features of sleep and activity, combined with baseline clinical variables, could support daily morning triage for acute suicide risk in psychiatric inpatients admitted to a closed ward.

**Objective:**

This exploratory pilot study evaluated whether wearable-derived sleep and activity features could complement baseline clinical variables for daily morning risk triage among psychiatric inpatients in a closed ward.

**Methods:**

We conducted a prospective observational pilot study of 87 enrolled psychiatric inpatients. Of these, 84 had valid Columbia-Suicide Severity Rating Scale (C-SSRS) assessments, and 69 contributed 151 assessment-linked records with both valid C-SSRS labels and temporally aligned wearable-derived sleep/activity features. Participants wore Fitbit Sense devices throughout hospitalization to collect passive sleep and activity data. Physical activity was summarized into 14 non-overlapping 2-hour windows spanning the previous day and assessment morning, ending at the 10:00 AM C-SSRS assessment. These features were combined with baseline clinical variables, including demographics and baseline C-SSRS score, to develop an L1-penalized logistic regression (LASSO) model. Performance was evaluated using recall (sensitivity) and the F2-score.

**Results:**

The multimodal fusion model showed numerically higher recall than the conventional assessment model (0.560 vs 0.289) and a higher F2-score (0.548 vs 0.300). However, the 95% confidence intervals overlapped substantially across models; therefore, these findings should be interpreted as exploratory and hypothesis-generating rather than as confirmatory evidence of model superiority. The fusion model identified C-SSRS-positive records from patients with low admission scores using wearable-derived activity-pattern features, particularly blunted morning activity (08:00–10:00) and nocturnal hyperactivity (22:00–24:00).

**Conclusion:**

These exploratory findings suggest that wearable-derived sleep and activity features may provide complementary information for daily morning risk triage in psychiatric inpatients. Larger studies are needed to validate whether this approach can support routine clinical review beyond baseline clinical variables.

## Graphical Abstract

**Figure:**
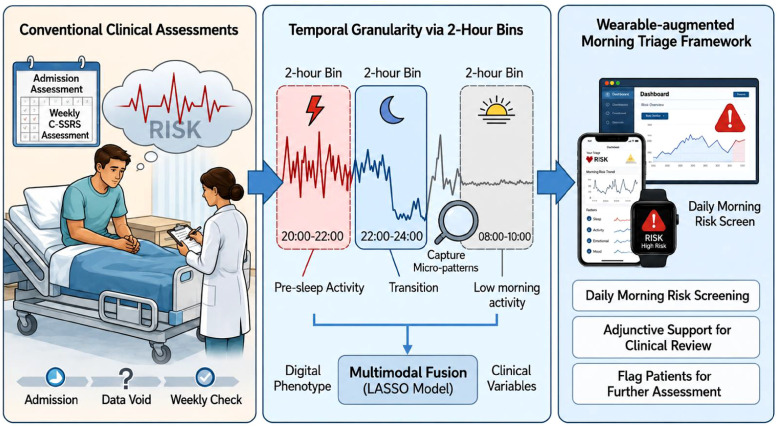
Schematic overview illustrating the transition from conventional intermittent clinical assessments to a dynamic, wearable-based multimodal monitoring framework for suicide risk.

## Introduction

Suicide remains one of the most critical concerns in the clinical care of patients with psychiatric disorders (1). Previous studies have shown that suicidal ideation and related behaviors occur with increased frequency during acute phases of severe mental illness, underscoring the heightened vulnerability of this population (2). It has been estimated that approximately 25% to 50% of patients with severe psychiatric conditions, such as bipolar disorder and schizophrenia, attempt suicide at some point during their illness course (3–5).

Although research specifically examining suicide attempts during psychiatric hospitalization is relatively limited compared to community samples, existing literature suggests that approximately 4% to 15% of inpatients attempt suicide or exhibit non-suicidal self-injury during admission (6). Accordingly, even within protected psychiatric wards—where intensive therapeutic interventions are provided for acutely ill patients—careful monitoring of suicide risk and the timely application of appropriate preventive strategies remain essential components of clinical practice (7).

Traditionally, suicide risk monitoring in psychiatric settings has relied primarily on detailed clinical interviews aimed at assessing suicidal ideation (1). To facilitate more structured and quantitative evaluation, clinician-rated instruments and patient self-report scales have also been widely employed (8). In addition, close clinical observation of individuals identified as high-risk (based on established factors such as a history of prior suicide attempts or a family history of suicide) has constituted a key approach to suicide risk management (7, 9).

However, these conventional methods possess inherent limitations. Interview-based assessments may not fully capture the rapidly fluctuating nature of suicidal ideation, particularly among acutely ill psychiatric inpatients who often experience marked emotional instability (10). Because suicidal thoughts may vary substantially over short intervals, moment-to-moment changes can be challenging to detect through periodic assessments alone (11). Moreover, continuous close observation by clinical staff may contribute to increased workload and fatigue, and monitoring gaps may arise during periods of reduced staffing, such as nights or weekends (12).

In response to these challenges, there has been growing interest in ecological momentary assessment (EMA) approaches; such real-time assessments demonstrate that suicidal thoughts can fluctuate substantially over short periods, highlighting the need for temporally dense monitoring over isolated cross-sectional assessments (13, 14). More broadly, digital phenotyping research has examined multiple candidate signals, including speech characteristics, smartphone-derived behavioral data, electrodermal activity, sleep variability, facial expressions, language use, and multimodal machine-learning models, to improve suicide-risk detection in naturalistic settings (15–24).

Importantly, much of this prior work has been conducted outside psychiatric inpatient wards. For example, one study followed young adults after an emergency-department visit for recent suicidal ideation or suicide attempts, using mobile-based EMA four times daily and Fitbit-derived passive sensing over 8 weeks (23). Another study examined high-risk adolescents during the month following discharge from acute psychiatric care using daily sleep diaries, actigraphy, and repeated assessments of suicidal thinking (24). Furthermore, in outpatient and community-based contexts, passive smartphone monitoring has been used to estimate short-term suicide-risk events from behavioral profiles such as activity, mobility, time spent at home, and app-use patterns (15). While these studies suggest that temporally dense digital monitoring may provide relevant information across various transitional and outpatient settings, such environments differ substantially from an acute closed psychiatric ward.

Among these digital signals, sleep and activity patterns have emerged as clinically relevant but heterogeneous predictors of suicide risk. Prior EMA and sleep studies have linked disturbed sleep, sleep variability, insomnia, nightmares, and altered circadian or rest-activity rhythms with suicidal ideation or suicide-risk states (13, 19). However, passive sensing has not consistently outperformed self-reported EMA or diary-based assessments. For instance, self-reported EMA features have been shown to predict next-day suicidal ideation with good accuracy, whereas passive wearable-derived sleep, activity, and resting-heart-rate features alone showed poor discrimination; adding passive sensing did not improve the EMA-only model (23). Similarly, diary-based sleep indices—including longer sleep onset latency, nightmares, and pre-sleep rumination—have successfully predicted next-day suicidal thinking, whereas actigraphy-derived sleep indices were less consistently associated with risk (24). A recent systematic review also concluded that passive sensing studies generally show lower and more heterogeneous predictive performance than active self-report data, leaving their incremental value uncertain (25). Thus, wearable-derived sleep and activity features should currently be interpreted as complementary passive sensing markers rather than replacements for self-report EMA or clinician-administered suicide-risk assessments.

Nevertheless, important implementation gaps remain. As noted, most prior EMA and passive sensing studies have focused on non-inpatient or transitional cohorts, and many have used self-reported suicidal ideation as the primary prediction outcome. Considerably less is known about whether passive wearable-derived sleep and activity features can support routine clinical risk triage in acute closed psychiatric wards. In these settings, suicide-risk assessment is already embedded in intensive staff supervision, and administering frequent self-report EMAs may impose an undue burden on both patients and clinicians. Furthermore, even within inpatient populations, previous wearable research has predominantly emphasized feasibility and acceptability over assessment-linked risk prediction; for instance, while physiological monitors have been successfully deployed among suicidal adolescent inpatients, predictive modeling was not the primary objective (26).

To address this specific implementation gap, the present study investigates whether low-burden, wearable-derived sleep and activity features—temporally aligned with routine morning Columbia-Suicide Severity Rating Scale (C-SSRS) assessments—can complement static baseline clinical variables for daily risk triage in an acute closed psychiatric ward. By summarizing activity patterns into non-overlapping 2-hour windows and integrating these features with baseline clinical information, we aimed to explore whether passive wearable monitoring can provide complementary information for identifying C-SSRS-defined acute suicide-risk states among psychiatric inpatients.

## Methods

### Study design and setting

We conducted a prospective observational exploratory study within a single-center closed psychiatric ward to evaluate the feasibility of collecting wearable-derived sleep/activity data and to explore their potential relevance for assessment-linked suicide-risk triage.

This study was approved by the Institutional Review Board of Korea University (IRB No. 2022GR0511) and conducted in accordance with the Declaration of Helsinki. Written informed consent was obtained from all participants or their legal guardians.

The clinical staff administering the Columbia-Suicide Severity Rating Scale (C-SSRS) (27) were blinded to all wearable-derived data and model predictions throughout the study period.

### Participants and recruitment

Of 169 psychiatric inpatients screened during the study period, 87 participants were enrolled and constituted the enrolled cohort. This enrolled cohort was used for baseline demographic and clinical summaries. Among the 87 enrolled participants, 84 participants had valid C-SSRS assessments and constituted the clinical assessment cohort. The predictive modeling dataset was subsequently constructed from 69 participants who had both valid C-SSRS labels and temporally aligned wearable-derived sleep/activity features, yielding 151 assessment-linked observation records for model training and evaluation. Eligible participants were adult psychiatric inpatients with an ICD-10 psychiatric diagnosis warranting admission to the closed ward and who were able to understand the study procedures. Although mood disorders and schizophrenia-spectrum disorders were the most common diagnostic categories, the enrolled cohort included a broader range of psychiatric diagnoses, as summarized in **Table 1**.

**Table 1:** Demographic and clinical characteristics of the enrolled cohort (N = 87).

Characteristic	Value
Total participants (N)	87
Age (years)	32.2 ± 18.2
Sex	
Male	25 (28.7%)
Female	62 (71.3%)
Length of Stay (Days)	23.2 ± 11.9
Primary diagnosis	
Mood Disorders	66 (75.9%)
Schizophrenia, Schizotypal, and Delusional Disorders	11 (12.6%)
Attention deficit hyperactivity disorder (ICD-10 F90.0)	3 (3.4%)
Neurotic, Stress-Related, and Somatoform Disorders	2 (2.3%)
Other	5 (5.7%)
Baseline C-SSRS Score	14.79 ± 7.09 (n=80)
Average Wear Time (Days)	16.0 ± 10.3

Values are presented as mean ± standard deviation (SD) for continuous variables and n (%) for categorical variables. Diagnostic categories were based on the primary recorded clinical diagnosis, and comorbid diagnoses were not double-counted. Attention deficit hyperactivity disorder corresponds to ICD-10 F90.0. The “Other” category included alcohol use disorder, subcortical vascular dementia, organic mood disorder, Alzheimer’s disease, and delirium. Baseline Week 0 C-SSRS scores were available for 80 participants. The predictive modeling dataset was constructed from 69 participants contributing 151 assessment-linked observation records.

### Clinical outcome measures and target labeling

Suicide risk was assessed using the Full Version of the C-SSRS, administered by trained clinical staff. The C-SSRS evaluates suicidal ideation and suicidal behavior, and the total score used in this study ranged from 0 to 25. A total score of 0 indicated that the patient denied all assessed suicidal thoughts or wishes, whereas scores greater than 0 indicated the presence of at least some suicidal ideation or related suicide-risk signal.

The C-SSRS was used in two distinct ways in this study. First, the initial C-SSRS assessment collected at admission was used as the baseline clinical suicide-risk variable in the conventional assessment model and the multimodal fusion model. Second, subsequent C-SSRS assessments, administered approximately weekly during hospitalization, were used as the outcome measure, or ground-truth label, for assessment-linked prediction. In this context, the term “ground-truth label” refers to the clinician-administered follow-up C-SSRS classification used as the reference outcome for model training and evaluation. Each assessment-linked record therefore paired wearable-derived sleep/activity features and baseline clinical variables with the corresponding follow-up C-SSRS outcome.

Because the distribution of follow-up C-SSRS scores was highly zero-inflated, we formulated the primary prediction task as a binary classification problem:

- Class 0 (Low Risk): C-SSRS score = 0.
- Class 1 (High Risk): C-SSRS score > 0.

This binary threshold was selected to identify whether any suicidal thoughts or wishes were present. In an acute closed-ward setting, even a non-zero C-SSRS score may require clinical attention, and the model was intended as an adjunctive screening tool to reduce missed-risk cases rather than as a standalone severity-grading instrument.

In the final constructed dataset used for model training (comprising 151 assessment-linked observation records from 69 unique patients with wearable data), this resulted in a highly imbalanced class distribution: 113 records (74.8%) for Class 0 and 38 records (25.2%) for Class 1. The 38 high-risk records were contributed by 22 unique patients (mean 1.73 records per patient), while the 113 low-risk records originated from 59 unique patients (mean 1.92 records per patient). Notably, 12 patients contributed records to both classes across different assessment time points, reflecting the dynamic and fluctuating nature of suicide risk during hospitalization. A histogram of the follow-up C-SSRS score distribution is provided in **Supplementary Figure 1**.

### Wearable sensor data collection

Participants wore a Fitbit Sense (Fitbit LLC, San Francisco, CA) on their non-dominant wrist for the duration of hospitalization (mean wear time: 16.0 ± 10.3 days). While consumer-grade, this device was chosen to evaluate real-world clinical applicability (28). For assessment-linked modeling, wearable-derived features were aligned to a 10:00 AM reference point on the day of each C-SSRS assessment, corresponding to the routine morning assessment workflow in the ward.

### Sleep feature engineering

Sleep data were obtained from Fitbit sleep stage tracking. Only records satisfying all three of the following conditions were retained: (a) Sleep type = “stages” (full sleep stage data available) (b) Main sleep = True (primary sleep episode), (c) Sleep onset (startTime) occurred before the date of sleep (dateOfSleep), i.e., the participant fell asleep before midnight.

Seven sleep features were extracted per night:

- deep_minutes: Minutes in the deep sleep stage.
- wake_minutes: Minutes awake during sleep period.
- light_minutes: Minutes in the light sleep stage.
- rem_minutes: Minutes in the REM sleep stage.
- startTime: Minutes before midnight at which sleep onset occurred (dateOfSleep – startTime, in minutes).
- endTime: Minutes after midnight at which sleep ended (time of awakening) (endTime – dateOfSleep, in minutes).
- efficiency: Sleep efficiency (%) as reported by Fitbit.

Missing sleep dates were interpolated within each patient’s time series to preserve the temporal continuity of nightly sleep features. Linear interpolation was applied in both directions for short gaps, with a 3-day buffer around the available sleep-data range, and interpolated values were rounded to integers. This approach was used to reduce the loss of assessment-linked observations caused by intermittent missing sleep-stage data in this exploratory pilot dataset.

### Feature engineering and binning strategy

To develop an EMA framework, wearable data were processed into non-overlapping 2-hour segments. This window size aligns with Kleitman’s Basic Rest-Activity Cycle (29) and captures the duration of acute suicidal impulses (approx. 1.4 hours) without diluting transient agitation signals (30).

Each 2-hour activity feature represents the patient’s normalized activity level during a specific 120-minute period. To reduce the influence of brief noise or isolated movement, minute-level movement data were converted into active versus inactive minutes, short inactive gaps within activity periods were smoothed, and the resulting value was normalized within each 2-hour window. Thus, each 2-hour bin can be interpreted as a normalized activity-density value for that specific time period:

#### Binarization

Minute-level movement ≥100 cm was labeled as “Movement (1), “ while minimal activity (< 100 cm) was labeled “Stationary (0).” This threshold was intended to distinguish meaningful ambulation from minimal movement or brief within-bed motion.

#### Temporal smoothing

A “10-minute gap-filling” rule was applied to treat short stationary intervals (< 10 min) between movements as continuous activity bouts.

#### Normalization

The corrected counts were normalized to a 0–1 scale within each 120-minute bin.

### Variable nomenclature

For interpretability, variables were systematically named using the format [Metric]_[Day]_[Time]. For example, Act_T_08–10 denotes activity density between 08:00 and 10:00 on the index day (Today), while Act_Y_22–24 refers to the preceding day (Yesterday). This distinction accounts for sleep-wake cycles extending past midnight.

### Feature sets

**Figure 1:**
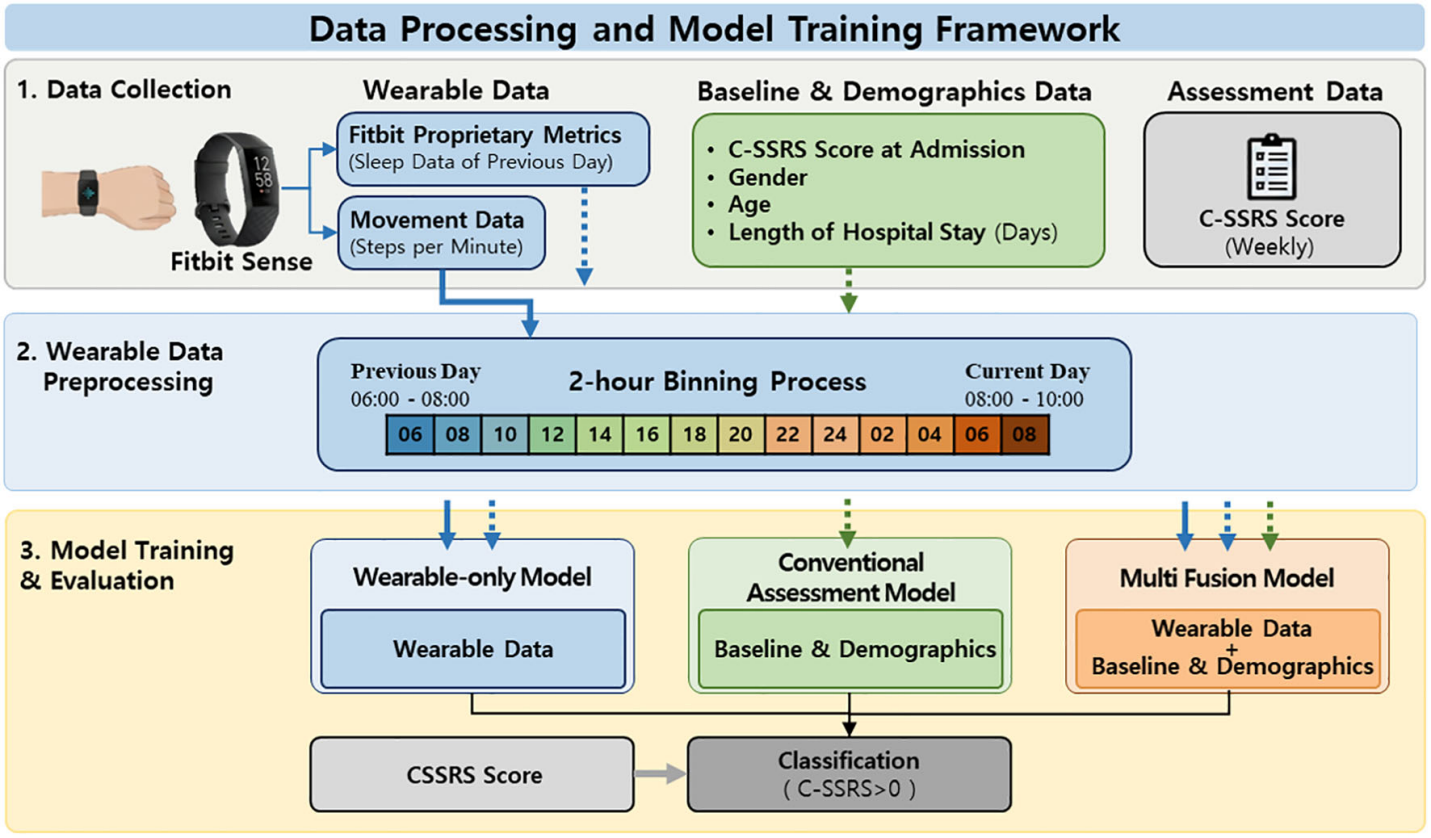
Workflow of wearable-augmented morning triage in an acute psychiatric ward. Continuous wearable-derived sleep and activity data collected via Fitbit Sense were aggregated with baseline clinical variables. The activity-binning window spanned from 06:00 on the previous day to 10:00 on the current assessment day, yielding 14 activity-related 2-hour features aligned with the 10:00 AM C-SSRS assessment.

To systematically compare model performance, we defined three distinct input feature sets as illustrated in **Figure 1**:

#### Wearable-only model

Included 21 features derived entirely from wearable devices. This comprised seven sleep-related variables (deep sleep minutes, wake minutes, light sleep minutes, REM sleep minutes, start time, end time, and sleep efficiency) and 14 activity-related features representing non-overlapping 2-hour intervals across the assessment-aligned 28-hour window.

#### Conventional assessment model

Included four demographic and baseline clinical features: sex, age, length of hospital stay (hospitalization day), and baseline suicide risk (first C-SSRS value).

#### Multimodal fusion model

Integrated all 25 features from both the wearable and clinical datasets to evaluate the synergistic effect of multimodal data.

### Classification algorithm

For the predictive modeling, we employed an L1-penalized Logistic Regression (LASSO) algorithm (penalty=‘l1’, C = 10, solver=‘liblinear’). The LASSO approach was specifically chosen for its suitability for handling complex multimodal data within a relatively small pilot sample (N = 69 patients, 151 observation records) (31). The model inherently performs feature selection by applying an L1 penalty, shrinking the coefficients of less relevant variables to zero. This mechanism effectively reduces data dimensionality and mitigates the risk of overfitting. Furthermore, the linear nature of the LASSO algorithm provides high clinical interpretability, allowing for a clear understanding of which specific variables most strongly drive suicide risk prediction. To justify this choice of architecture, we benchmarked LASSO against four alternative machine learning algorithms (Random Forest, XGBoost, KNN, and SVM), the comprehensive performance metrics and fixed structural hyperparameters of which are detailed in **Supplementary Tables 1**, **2**, respectively.

### Cross-validation strategy

To account for the repeated-measures structure of the data—where multiple assessment points exist per patient—model performance was evaluated using a patient-level repeated stratified K-fold cross-validation strategy. The dataset was split into five folds with 1, 000 repetitions using a fixed random seed of 42, resulting in 5, 000 validation iterations. Stratification was based on patient-level binary labels, assigned as 0 if all C-SSRS state values for a given patient were 0, and 1 otherwise. The analysis examined predictive patterns across assessment-linked records, while accounting for repeated records from the same patient, rather than explicitly modeling within-person change from each patient’s own prior baseline. All records from the same patient were assigned entirely to either the training or validation fold to prevent patient-level leakage.

Sleep-feature interpolation was applied within patient-level time series to preserve temporal continuity of intermittently missing sleep-stage data. Given the limited size of the pilot dataset, excluding interpolated validation records would have further reduced the number of usable assessment-linked observations and increased performance variability. We therefore interpret the results cautiously and identify fold-internal imputation and sensitivity analyses excluding interpolated sleep records as priorities for future validation studies. To prioritize safety in suicide risk detection, model evaluation focused on recall and F2-score, which weights recall higher than precision and is therefore aligned with minimizing clinically critical false negatives (32).

As a sensitivity analysis addressing hyperparameter selection, we additionally performed nested patient-level cross-validation for the L1-penalized logistic regression model. In the inner loop, the regularization strength was selected using grid search over C ∈ {0.01, 0.03, 0.1, 0.3, 1, 3, 10, 30, 100}, with F2-score as the optimization criterion. The selected model was then refitted on the full outer training fold and evaluated on the corresponding outer validation fold. This analysis was used to assess whether the original fixed setting of C = 10 materially influenced the reported performance estimates. To further characterize the stability of model performance, we additionally retained validation-fold performance metrics for the LASSO models across all 5, 000 fold-iterations generated by repeated patient-level stratified 5-fold cross-validation. For each feature set, fold-level accuracy, precision, recall, and F2-score were summarized using the mean, standard deviation, variance, minimum–maximum range, and empirical 2.5th–97.5th percentile interval (**Supplementary Table 3**). These fold-level summaries were intended to describe empirical sampling variability across validation folds rather than to provide independent inferential estimates, because repeated cross-validation folds are not statistically independent.

### Software and implementation

All computational analyses were conducted using Python (version 3.8). Data processing was performed using pandas and numpy, while machine learning pipelines, cross-validation, and scaling were implemented with scikit-learn. Model explanations and feature importance were derived using the SHAP library (utilizing LinearExplainer and summary_plot). Visualizations were generated using matplotlib and seaborn. To improve reproducibility across all procedures, random seeds were fixed at 42.

### Missing data handling

Baseline Week 0 C-SSRS scores were available for 80 participants among those with valid C-SSRS assessment data. For four participants without Week 0 assessment, the earliest available C-SSRS score during hospitalization was used as the baseline value for clinical characterization and modeling when applicable. The predictive modeling dataset was restricted to 69 participants who had both valid C-SSRS labels and temporally aligned wearable-derived sleep/activity features, resulting in 151 assessment-linked observation records. Missing sleep data were handled using the interpolation method described above.

## Results

### Feasibility and compliance

**Figure 2:**
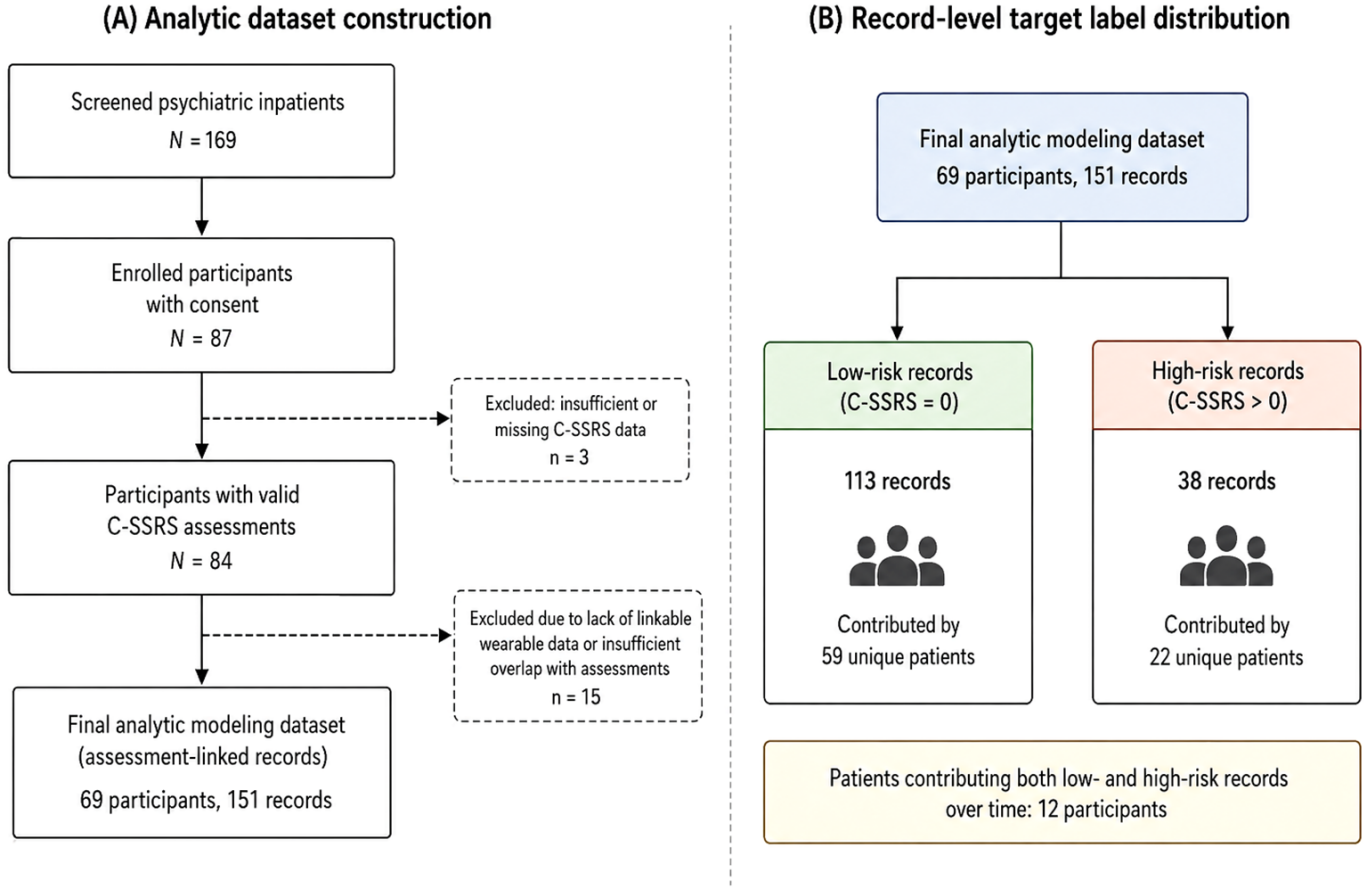
Participant enrollment and transition from patient-level cohorts to the record-level predictive modeling dataset. **(A)** Analytic dataset construction flow. Of 169 screened psychiatric inpatients, 87 participants were enrolled and included in the baseline demographic and clinical summary cohort. Among them, 84 participants had valid Columbia-Suicide Severity Rating Scale (C-SSRS) assessments. The final analytic modeling dataset was constructed from 69 participants with both valid C-SSRS labels and temporally aligned wearable-derived sleep/activity features, yielding 151 assessment-linked observation records. **(B)** Record-level target label distribution in the final analytic modeling dataset. Low-risk records were defined as C-SSRS = 0 and high-risk records as C-SSRS > 0; the dataset included 113 low-risk records and 38 high-risk records, contributed by 59 and 22 unique participants, respectively, with 12 participants contributing records to both classes over time.

Of the 169 screened patients, 87 participants were enrolled and used for baseline descriptive summaries. Among them, 84 participants had valid C-SSRS assessments (**Figure 2**). The predictive modeling dataset included 69 participants with valid C-SSRS labels and temporally aligned wearable-derived sleep/activity features, yielding 151 assessment-linked observation records. The mean device wear time among enrolled participants was 16.0 days (SD 10.3). However, detailed day-by-day adherence patterns, reasons for non-wear, and device-level signal-quality metrics were not systematically analyzed in this exploratory pilot study. The baseline characteristics of all 87 enrolled participants are summarized in **Table 1**. Primary diagnoses were classified according to the primary recorded clinical diagnosis, and comorbid diagnoses were not double-counted. The mean age was 32.2 years (SD 18.2), and 71.3% (62/87) were female. Mood disorders were the primary diagnosis for 75.9% (66/87) of the sample, indicating that mood-disorder presentations were the most common diagnostic category in the enrolled cohort. The average length of stay was approximately 23 days, allowing repeated assessment-linked observations to be collected during hospitalization.

### Model performance: capturing latent risk

Model performance was evaluated using standard binary-classification metrics. Accuracy indicates the proportion of all records correctly classified. Sensitivity, also referred to as recall, indicates the proportion of C-SSRS-positive records correctly identified by the model. Specificity indicates the proportion of C-SSRS-negative records correctly identified. Precision indicates the proportion of model-positive predictions that were truly C-SSRS-positive. The F2-score summarizes precision and recall while assigning greater weight to recall; this metric was emphasized because the model was intended as an adjunctive screening tool in which reducing missed-risk cases was prioritized.

As shown in **Table 2**, the conventional assessment model showed limited sensitivity for high-risk records. The overall discriminative ability and threshold trade-offs for each distinct feature configuration are further illustrated via the Receiver Operating Characteristic (ROC) curves in **Supplementary Figure 2**. The multimodal fusion model showed numerically higher recall than the conventional assessment model (0.560 vs. 0.289) and a higher F2-score (0.548 vs. 0.300), suggesting that wearable-derived sleep and activity features may add sensitivity-oriented information beyond baseline clinical assessment. However, the empirical 95% intervals overlapped substantially across models, and the fold-level analysis showed considerable variability in recall and F2-score. Therefore, these findings should be interpreted as exploratory evidence supporting the potential utility of multimodal fusion for reducing missed high-risk cases, rather than as definitive evidence of model superiority.

**Table 2:** Exploratory performance estimates of conventional, wearable-only, and multimodal fusion models for C-SSRS-defined risk classification.

Model	Accuracy	Recall	Precision	F2-score
Wearable-only Model	0.710 (0.559-0.848)	0.288 (0.000-0.667)	0.402 (0.000-1.000)	0.295 (0.000-0.625)
Conventional Assessment Model	0.743 (0.545-0.906)	0.289 (0.000-0.750)	0.529 (0.000-1.000)	0.300 (0.000-0.729)
Multimodal Fusion Model	0.773 (0.600-0.912)	0.560 (0.143-0.875)	0.556 (0.111-1.000)	0.548 (0.151-0.833)

Values are presented as mean (95% CI). The models used L1-penalized logistic regression and were evaluated using patient-level repeated stratified K-fold cross-validation. Confidence intervals overlapped substantially across models; therefore, between-model differences should be interpreted as exploratory rather than confirmatory.

For the multimodal fusion model, recall and F2-score showed higher mean values than the wearable-only and conventional assessment models, but their fold-level ranges remained wide. This indicates that although the multimodal fusion model improved average sensitivity-oriented performance, performance on any individual validation fold may still vary substantially.

A nested cross-validation sensitivity analysis further showed that inner-loop tuning of the LASSO regularization parameter did not materially alter model performance (**Supplementary Table 4**). The empirical stability of this parameterization is further supported by the inner-loop regularization strength selection frequencies summarized in **Supplementary Table 5**. For the multimodal fusion model, nested cross-validation yielded a recall of 0.552 and an F2-score of 0.544, which were highly similar to the original fixed-C results of 0.560 and 0.548, respectively.

To improve model interpretability, standardized LASSO logistic regression coefficients for the multimodal fusion model are provided in **Supplementary Table 6**. These coefficients indicate the direction and relative magnitude of each predictor’s association with C-SSRS-positive status after feature standardization. Because the analysis was observational and predictive, the coefficients should be interpreted as model-based associations rather than causal effects.

### Reclassification of hidden risk

**Figure 3:**
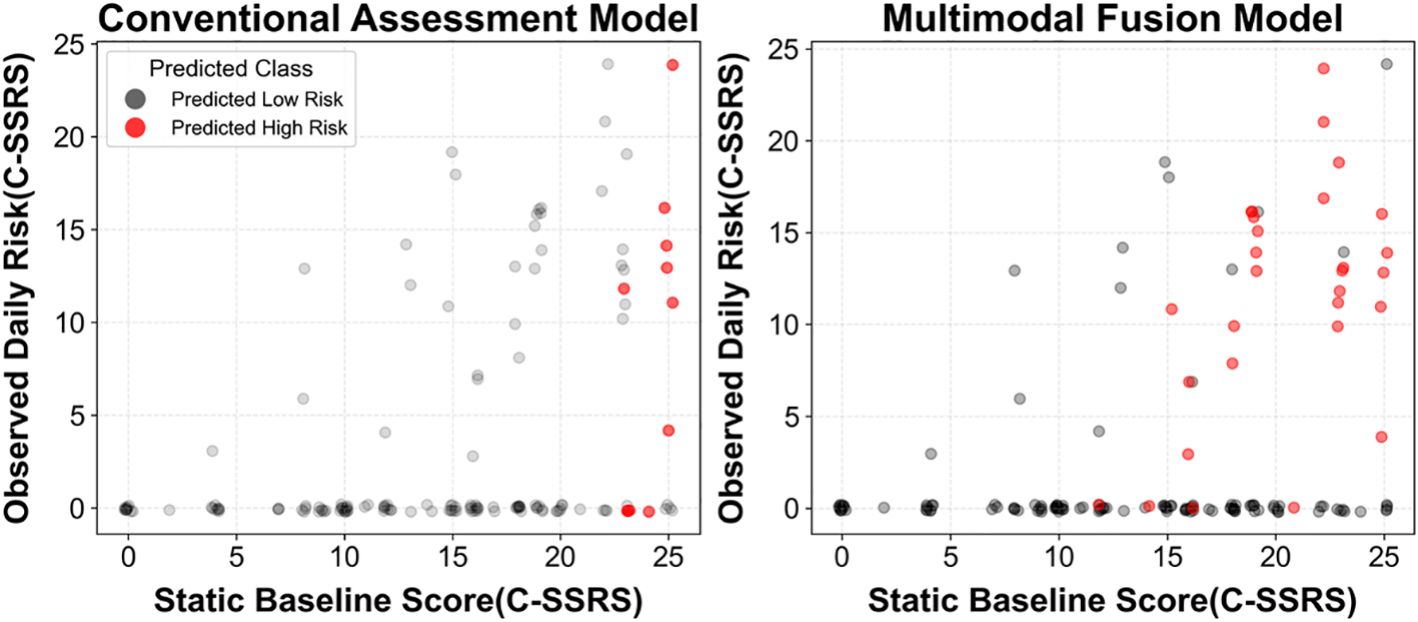
Comparison of suicide risk detection performance between models. The conventional assessment model (left) exhibits a rigid dependence on extreme baseline scores, predominantly classifying instances as high risk only when admission scores are high. Consequently, it fails to detect acute risk (Y-axis > 10) in patients with baseline scores in the 15–20 range. In contrast, the multimodal fusion model (right) leverages learned physiological parameters to identify these high-risk instances even among patients with moderate baseline scores (X-axis: 15–20), effectively capturing acute distress that would otherwise remain undetected by traditional static assessments.

The potential clinical relevance of this approach is visualized in **Figure 3**, which shows how the fusion model reclassifies patients whose risk was initially obscured. The conventional assessment model’s high-risk classifications were primarily associated with high baseline (admission) severity. This pattern suggested a potential for reduced sensitivity in patients within the 15–20 baseline score range, where acute physiological changes may not have been fully captured by static clinical indicators alone. In contrast, the multimodal fusion model demonstrated an enhanced capacity to identify high-risk status (C-SSRS > 15) even within this intermediate baseline. By incorporating wearable-derived features, the model’s parameters were able to recognize subtle warning signs, thereby offering an adjunctive decision-support approach for patients whose latent risk might not be fully indicated by initial admission scores.

### Digital phenotype interpretation

**Figure 4:**
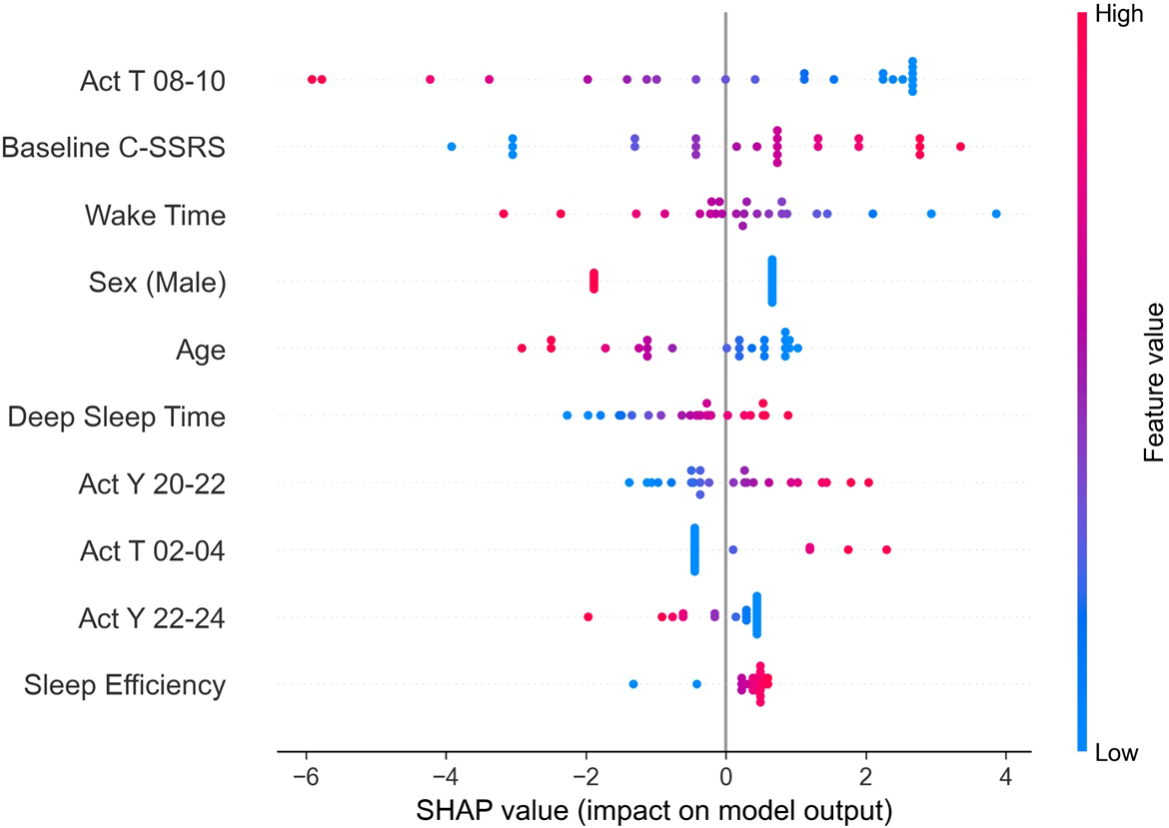
SHAP-based interpretation of the multimodal fusion model. The figure shows the top 10 features contributing to model predictions in the multimodal fusion model (L1-penalized logistic regression). Features are ranked by mean absolute SHAP value, with higher-ranked features having greater overall influence on the model’s predictions. Each point represents an assessment-linked observation record. The horizontal axis shows the SHAP value: positive values indicate that the feature pushed the model toward a higher predicted probability of C-SSRS-positive status, whereas negative values indicate a lower predicted probability. Color indicates the actual feature value, with red representing higher values and blue representing lower values.

We used SHAP analysis (**Figure 4**) to interpret the multimodal fusion model. SHAP is a model-explanation method that estimates how much each feature contributes to a model prediction for each observation (33). Features with larger mean absolute SHAP values had greater overall influence on the model’s predictions. In the SHAP summary plot, the horizontal position indicates whether a feature pushed the model toward a higher or lower predicted probability of C-SSRS-positive status, whereas the color indicates the actual feature value, with red representing higher values and blue representing lower values. The highest-ranked features included morning activity, sleep timing, sleep-stage variables, and evening activity windows. Among these, Act_T_08–10 showed the largest mean absolute SHAP value, indicating that activity in the 08:00–10:00 window immediately preceding the C-SSRS assessment had the greatest overall influence on model predictions. Specifically, as shown by the concentration of low-value (blue) points on the positive side of the SHAP axis, it was a reduction or blunting of physical activity during this morning window that strongly drove the model toward predicting a high suicide-risk state.

## Discussion

In this exploratory pilot study, the multimodal fusion model showed numerically higher sensitivity-oriented performance than the conventional baseline assessment model. However, this finding should be interpreted in light of the temporal structure of the data. Wearable-derived sleep and activity features were extracted from the period immediately preceding the C-SSRS assessment, whereas the conventional clinical model included baseline demographic and clinical variables. Therefore, the observed difference may partly reflect the advantage of temporally proximal information rather than the intrinsic superiority of passive physiological sensing over questionnaire-based assessment. Moreover, the confidence intervals overlapped substantially across models; therefore, these findings should be interpreted as hypothesis-generating rather than as confirmatory evidence of model superiority.

Prior studies have linked suicide risk with sleep disturbance, insomnia, sleep variability, nocturnal wakefulness, circadian disruption, and altered psychomotor activity (13, 19, 24, 34–36). These findings support the hypothesis that sleep and activity patterns may contain clinically relevant information related to short-term suicide risk. However, much of the existing evidence has been derived from outpatient, community, or young adult samples (15, 23, 24), and several studies have relied on self-report EMA or broader daily-level passive sensing rather than assessment-aligned inpatient monitoring (23–25). Importantly, prior work comparing active survey EMA with passive sensing suggests that passive wearable data should not be assumed to be superior to direct self-report assessment. Rather, wearable-derived sleep and activity features may provide complementary, low-burden information when frequent clinician-administered or self-report assessments are difficult to perform.

The present study extends this literature in three ways. First, it examined wearable-derived sleep and activity features in acutely hospitalized psychiatric inpatients in a closed ward, where medication use, restricted mobility, fixed routines, and staff observation may alter the interpretation of digital phenotyping signals. Second, it used assessment-aligned activity features, including 14 non-overlapping 2-hour windows ending at the routine 10:00 AM C-SSRS assessment point, rather than relying only on daily aggregate activity. Third, it evaluated whether these passive wearable-derived features provided complementary information when combined with baseline clinical variables. Therefore, our findings should be interpreted as exploratory evidence that sleep and activity signals may add contextual information for daily morning triage in a structured inpatient environment, rather than as evidence that wearable monitoring is superior to repeated clinical or self-report assessment.

The SHAP-ranked predictors provide a clinically interpretable hypothesis regarding why specific sleep and activity features may have contributed to model predictions. Among the activity variables, Act_T_08–10 showed the largest contribution. This window immediately preceded the routine 10:00 AM C-SSRS assessment and may therefore capture the patient’s most proximal morning behavioral state. In a closed psychiatric ward, activity during this period may reflect morning activation, engagement in ward routines, residual sleep inertia, delayed sleep-wake rhythm, or psychomotor slowing shortly before clinical evaluation. Reduced activity in this window may therefore indicate impaired morning engagement, psychomotor retardation, or disrupted circadian activation (37).

By contrast, Act_T_06–08 may have been less informative because this earlier period overlaps with early awakening, staff checks, medication routines, and the transition into scheduled ward activities (38, 39). Activity during this time may be more strongly shaped by institutional routines and sleep termination than by spontaneous patient behavior. Therefore, the later 08:00–10:00 window may better capture individual differences in morning engagement after the ward day has begun. These findings underscore that the relationship between activity and suicide risk is complex and non-linear. Consistent with recent studies emphasizing that variability may be a more critical predictor than mean activity levels alone (19, 40), our 2-hour binning approach proved highly effective in capturing non-linear shifts and patient-specific physiological variability that daily aggregated data might obscure.

Other influential variables also appeared to reflect sleep-wake and circadian patterns. Specifically, our SHAP analysis identified earlier wake time (ranked 3rd) as a prominent predictor. In the acute psychiatric ward context, earlier wake times serve as an objective proxy for terminal insomnia, a well-recognized risk factor for morning-hour suicidal distress and subsequent behavioral crises (34, 41). Longer deep sleep duration should be interpreted cautiously in the inpatient context, because it may reflect sedative medication exposure, prolonged immobility rather than restorative sleep alone. Furthermore, evening activity features may capture pre-sleep agitation or disruption in the evening-to-night transition (42, 43). Notably, classical clinical observations suggest that the risk of suicidal behavior may acutely increase during the recovery phase of depression, when energy levels begin to return and previously inhibited activity becomes reactivated (37). Capturing these abrupt shifts or inflection points from psychomotor retardation to heightened agitation is critical. Taken together, these findings suggest that the timing, transition, and variability of sleep and activity patterns, rather than total activity level alone, are highly relevant for suicide-risk triage in this setting.

From a clinical workflow perspective, the value of wearable-derived monitoring is not that it replaces direct suicide-risk assessment, but that it may provide passive and less disclosure-dependent information to support clinical review. In the closed-ward setting, C-SSRS assessments are generally performed at approximately weekly intervals because of staffing and workload constraints, and even this schedule can be burdensome in routine care. In addition, some patients may deny or underreport suicidal ideation or self-harm-related behavior during structured questioning. Wearable-derived sleep and activity features may therefore help identify behavioral or circadian patterns that warrant closer attention, particularly when frequent clinician-administered assessment or unsupervised self-report EMA is impractical or unreliable. By providing clinicians with a “Wearable-Augmented Morning Triage, “ this adjunctive monitoring framework allows for more focused interventions among patients whose digital phenotypes signal an impending crisis, even when their baseline clinical scores appear stable.

Beyond the inpatient setting, the present findings may have implications for future outpatient or community-based monitoring strategies, as consumer-grade wearable devices are already widely used in daily life. Passive sleep and activity features could potentially support longitudinal follow-up between clinical visits, particularly when frequent self-report assessment is burdensome or incomplete. Nevertheless, because the present study was conducted in a single closed psychiatric ward, such broader applications remain speculative and require prospective validation in outpatient and community samples.

### Limitations

Despite its strengths, this study has several limitations. As a single-center pilot study with 87 enrolled participants and 69 participants contributing 151 assessment-linked observation records to the predictive modeling dataset, the generalizability of the results is limited. The use of proprietary Fitbit algorithms for sleep stage classification may introduce bias, and future studies should aim to utilize raw accelerometry and heart rate variability data (28, 44). Additionally, although the multimodal fusion model showed numerically higher average recall and F2-score than the conventional assessment model, fold-level variability remained substantial, particularly for sensitivity-oriented metrics. This reflects the limited number of high-risk observations per validation fold and indicates that model performance on a single new dataset may be unstable. The wide and overlapping confidence intervals across models further indicate that between-model differences should be interpreted cautiously as exploratory findings rather than confirmatory evidence of model superiority. Further refinement is also needed to reduce false-positive screening signals and minimize unnecessary clinical burden among staff.

Because the model was trained on assessment-linked records, unequal numbers of C-SSRS assessments across patients may have influenced model estimation. Although patient-level grouped cross-validation was used to avoid patient-level leakage, future validation studies should prospectively report assessment frequency and evaluate patient-weighted modeling strategies. In addition, this study did not include survey EMA measures of sleep, activity, mood, or suicidal ideation. Therefore, we could not directly compare passive wearable EMA with active self-report EMA or with more frequent clinician-administered C-SSRS assessments. Because wearable-derived features were temporally closer to the outcome C-SSRS assessment than baseline clinical variables, part of the observed model difference may be attributable to timing. Missing sleep-stage data were handled using interpolation within patient-level time series; although this approach preserved temporal continuity in the pilot dataset, it may still influence model performance estimates. Future studies with larger datasets should implement fold-internal imputation pipelines and perform sensitivity analyses excluding interpolated sleep records to assess the robustness of sleep-derived predictors.

## Conclusion

This exploratory pilot study suggests that wearable-derived sleep and activity features may provide complementary information for daily morning suicide-risk triage in psychiatric inpatients. However, the findings require confirmation in larger prospective studies with external validation before clinical implementation.

Critically, the application of a 2-hour binning strategy revealed a distinct physiological signature characterized by heightened pre-sleep activity, a distinct evening-to-nocturnal transition, and severe physiological inertia in the morning. These findings suggest that the specific timing and transition of circadian disruptions, rather than activity levels in isolation, are vital indicators of acute distress. This supports the potential of a “Wearable-Augmented Morning Triage System”, which could serve as an objective, adjunctive safety net to alert clinicians to physiological warning signs before behavioral crises occur.

While these results are promising, they represent a formative step. Future large-scale, multi-center studies are necessary to validate these passive wearable-derived features across diverse patient populations and to formalize their integration into daily clinical decision support systems.

## Data Availability

The raw data supporting the conclusions of this article will be made available by the authors, without undue reservation.
