# Supplementary material for: Use of ecological momentary assessment via wearable devices for detecting acute suicide risk in psychiatric inpatients
Source: Front Psychiatry. 2026 Jul 1;17:1849140. doi: 10.3389/fpsyt.2026.1849140 (PMC13369052; doi:10.3389/fpsyt.2026.1849140)
Supplement: Supplementary file 1 [file SupplementaryFile1.docx]

**Supplementary Information**

*Use of Ecological Momentary Assessment via Wearable Devices for Detecting Acute Suicide Risk in Psychiatric Inpatients*

Yourack Lee, ByeongChang Jeong, Cheol E. Han, Hyun-Ghang Jeong


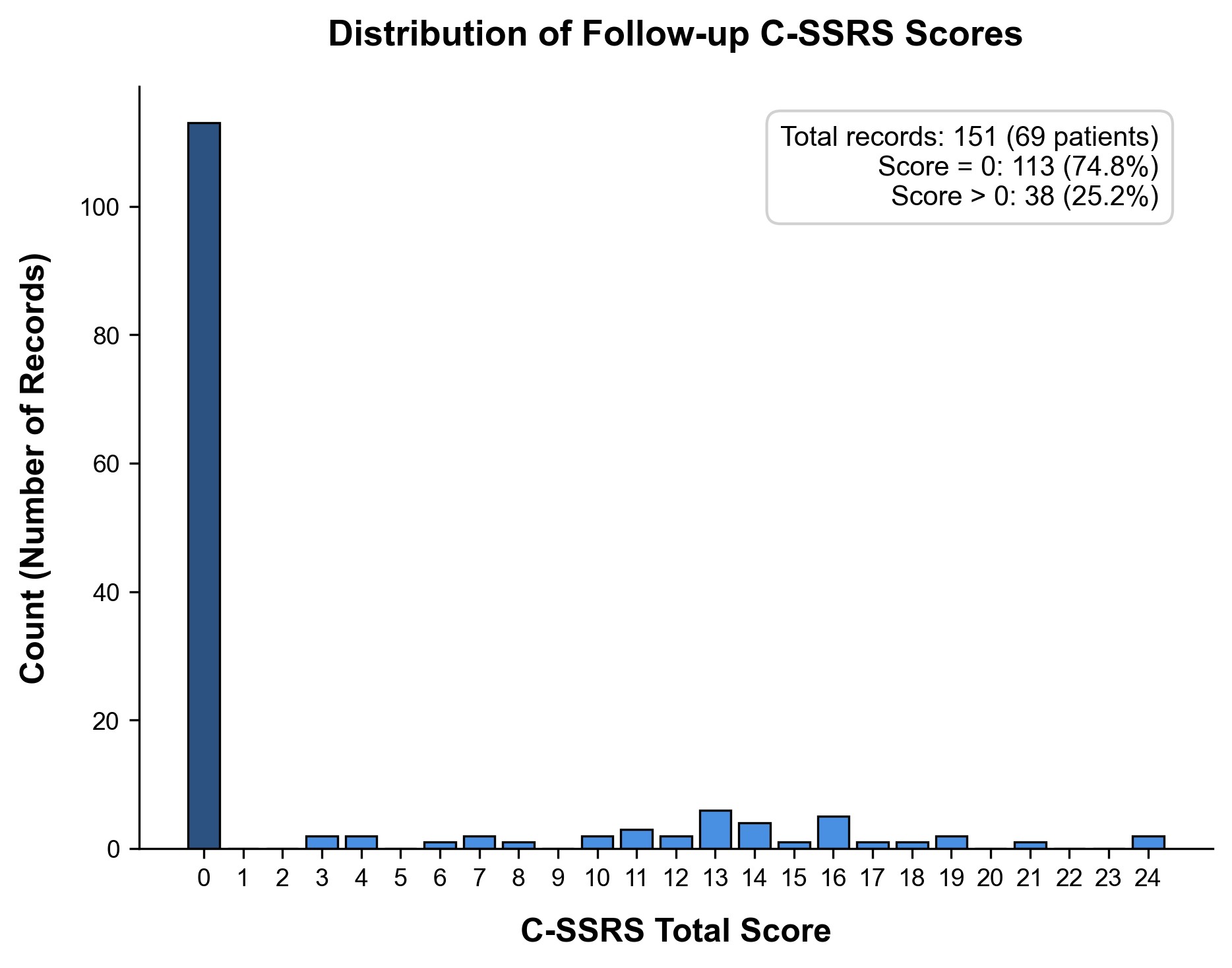


**Supplementary Figure S1. Distribution of follow-up C-SSRS total scores in the predictive modeling dataset.**
The histogram shows the distribution of C-SSRS total scores across 151 assessment-linked observation records from 69 unique patients. The distribution was highly zero-inflated, with 113 records (74.8%) having a C-SSRS score of 0 and 38 records (25.2%) having a score greater than 0. This distribution motivated the primary binary classification task used in the present exploratory pilot study.

**Supplementary Table S1.** Performance comparison of five classification algorithms across three input feature sets for suicide risk detection among psychiatric inpatients (N = 84, 151 observation records). Values are presented as the mean (95% confidence interval) derived from Repeated Stratified K-Fold Cross-Validation (five-fold × 1,000 repetitions = 5,000 iterations). L1-penalized logistic regression (LASSO) was selected as the primary model for the main analysis based on its superior F2-score and recall in the multimodal fusion configuration.

| **Algorithm** | **Metric** | **Wearable-only Model** | **Conventional Assessment Model** | **Multimodal Fusion Model** |
| --- | --- | --- | --- | --- |
| Random Forest | Accuracy | 0.748 (0.615–0.857) | 0.734 (0.562–0.885) | 0.755 (0.607–0.870) |
| Random Forest | Precision | 0.203 (0.000–1.000) | 0.468 (0.000–1.000) | 0.393 (0.000–1.000) |
| Random Forest | Recall | 0.029 (0.000–0.167) | 0.234 (0.000–0.667) | 0.085 (0.000–0.333) |
| Random Forest | F2-score | 0.035 (0.000–0.200) | 0.246 (0.000–0.638) | 0.098 (0.000–0.385) |
| XGBoost | Accuracy | 0.746 (0.607–0.857) | 0.740 (0.583–0.865) | 0.745 (0.607–0.857) |
| XGBoost | Precision | 0.011 (0.000–0.000) | 0.113 (0.000–1.000) | 0.092 (0.000–1.000) |
| XGBoost | Recall | 0.002 (0.000–0.000) | 0.041 (0.000–0.400) | 0.024 (0.000–0.250) |
| XGBoost | F2-score | 0.003 (0.000–0.000) | 0.044 (0.000–0.429) | 0.027 (0.000–0.294) |
| KNN | Accuracy | 0.692 (0.552–0.824) | 0.715 (0.519–0.879) | 0.723 (0.581–0.844) |
| KNN | Precision | 0.297 (0.000–1.000) | 0.446 (0.000–1.000) | 0.419 (0.000–1.000) |
| KNN | Recall | 0.146 (0.000–0.500) | 0.322 (0.000–0.750) | 0.231 (0.000–0.545) |
| KNN | F2-score | 0.156 (0.000–0.469) | 0.325 (0.000–0.714) | 0.247 (0.000–0.556) |
| SVM | Accuracy | 0.750 (0.618–0.857) | 0.719 (0.548–0.867) | 0.766 (0.621–0.879) |
| SVM | Precision | 0.177 (0.000–1.000) | 0.308 (0.000–1.000) | 0.575 (0.000–1.000) |
| SVM | Recall | 0.023 (0.000–0.143) | 0.158 (0.000–0.600) | 0.115 (0.000–0.375) |
| SVM | F2-score | 0.027 (0.000–0.172) | 0.165 (0.000–0.571) | 0.135 (0.000–0.429) |
| Lasso† | Accuracy | 0.710 (0.559–0.848) | 0.743 (0.545–0.906) | 0.773 (0.600–0.912) |
| Lasso† | Precision | 0.402 (0.000–1.000) | 0.529 (0.000–1.000) | 0.556 (0.111–1.000) |
| Lasso† | Recall | 0.288 (0.000–0.667) | 0.289 (0.000–0.750) | 0.560 (0.143–0.875) |
| Lasso† | F2-score | 0.295 (0.000–0.625) | 0.300 (0.000–0.729) | 0.548 (0.151–0.833) |

*Note: Models were trained using L1-penalized logistic regression (LASSO), Random Forest, XGBoost, K-Nearest Neighbors (KNN), and Support Vector Machine (SVM). Three feature sets were compared: the Wearable-only Model (21 wearable-derived features), Conventional Assessment Model (4 demographic/clinical features), and Multimodal Fusion Model (all 25 features). The LASSO model (†) was selected for the main analysis based on its superior F2-score performance. Evaluation was performed using patient-level grouped cross-validation to prevent data leakage.*


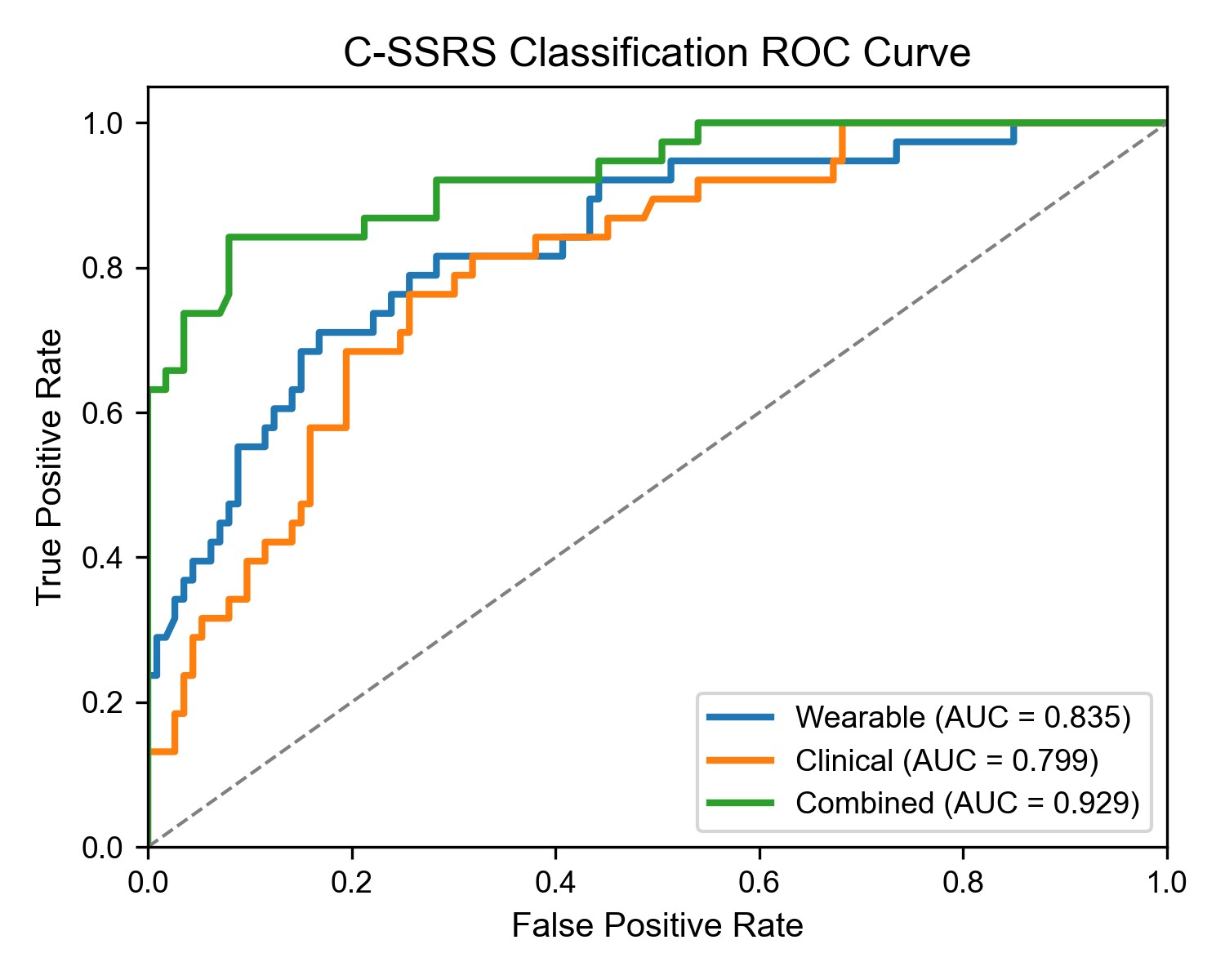


**Supplementary Figure S2.** Receiver operating characteristic (ROC) curve for the C-SSRS suicide risk prediction model. The ROC curve illustrates the trade-off between sensitivity (true positive rate) and 1-specificity (false positive rate) across different classification thresholds. The area under the curve (AUC) provides a summary measure of the model's overall discriminative ability for distinguishing between high-risk (C-SSRS > 0) and low-risk (C-SSRS = 0) patients.

**Supplementary Table S2.** Hyperparameters of the machine learning models used for C-SSRS suicide risk classification. All models were trained using the same cross-validation framework (Five-fold Repeated Stratified K-Fold, 1,000 repetitions) with patient-level grouped splitting. L1-penalized Logistic Regression (LASSO) was selected as the primary model for the main analysis based on its superior F2-score and clinical interpretability.

| **Model** | **Library** | **Hyperparameters** |
| --- | --- | --- |
| RandomForest Classifier | Scikit-learn | n_estimators=100, max_depth=5, min_samples_leaf=5, |
| XGBClassifier | XGBoost | n_estimators=500, max_depth=3, learning_rate=0.03, subsample=0.8, colsample_bytree=0.8, gamma=1, min_child_weight=5, reg_alpha=0.1, reg_lambda=1.0, eval_metric='logloss', early_stopping_rounds=10 |
| KNeighbors Classifier | Scikit-learn | n_neighbors=5 weights='uniform', metric='minkowski' |
| SVC | Scikit-learn | kernel='rbf', C=1.0, gamma='scale' |
| MLP (Keras Sequential) | TensorFlow / Keras | Layers=[Input, Dense(64,relu), Dense(32,relu), Dense(1,sigmoid)], optimizer='adam', loss='binary_crossentropy', epochs=50, batch_size=32, validation_split=0.2, EarlyStopping(patience=5, monitor='val_loss') |
| Logistic Regression (L1) | Scikit-learn | penalty='l1', C=10, solver='liblinear' |

*Note: L1-penalized Logistic Regression (LASSO) was selected as the primary classifier due to its inherent feature selection capability, high clinical interpretability, and superior F2-score in cross-validation. All other models were used as benchmarks for comparison (see Supplementary Table S1 for detailed performance metrics).*

**Supplementary Table S3. Fold-level variability of LASSO model performance across repeated patient-level stratified 5-fold cross-validation.**

| **Feature set** | **Metric** | **Mean** | **SD** | **Variance** | **Min** | **Q1** | **Median** | **Q3** | **Max** | **Empirical 95% interval** |
| --- | --- | --- | --- | --- | --- | --- | --- | --- | --- | --- |
| Wearable-only Model | Accuracy | 0.710 | 0.075 | 0.006 | 0.360 | 0.657 | 0.711 | 0.760 | 0.958 | 0.559–0.848 |
|  | Recall | 0.288 | 0.176 | 0.031 | 0.000 | 0.167 | 0.286 | 0.400 | 0.889 | 0.000–0.667 |
|  | Precision | 0.402 | 0.235 | 0.055 | 0.000 | 0.250 | 0.400 | 0.500 | 1.000 | 0.000–1.000 |
|  | F2-score | 0.295 | 0.168 | 0.028 | 0.000 | 0.172 | 0.294 | 0.417 | 0.833 | 0.000–0.625 |
| Conventional Assessment Model | Accuracy | 0.743 | 0.095 | 0.009 | 0.308 | 0.679 | 0.750 | 0.815 | 0.971 | 0.545–0.906 |
|  | Recall | 0.289 | 0.220 | 0.049 | 0.000 | 0.125 | 0.250 | 0.429 | 1.000 | 0.000–0.750 |
|  | Precision | 0.529 | 0.369 | 0.136 | 0.000 | 0.222 | 0.500 | 1.000 | 1.000 | 0.000–1.000 |
|  | F2-score | 0.300 | 0.215 | 0.046 | 0.000 | 0.139 | 0.287 | 0.455 | 0.962 | 0.000–0.729 |
| Multimodal Fusion Model | Accuracy | 0.773 | 0.081 | 0.007 | 0.440 | 0.722 | 0.778 | 0.833 | 1.000 | 0.600–0.912 |
|  | Recall | 0.560 | 0.202 | 0.041 | 0.000 | 0.429 | 0.571 | 0.714 | 1.000 | 0.143–0.875 |
|  | Precision | 0.556 | 0.218 | 0.048 | 0.000 | 0.400 | 0.556 | 0.700 | 1.000 | 0.111–1.000 |
|  | F2-score | 0.548 | 0.187 | 0.035 | 0.000 | 0.435 | 0.577 | 0.682 | 1.000 | 0.151–0.833 |

*Note: Values summarize test-set performance across 5,000 validation fold-iterations generated from repeated patient-level stratified 5-fold cross-validation with 1,000 repeats. Variance and range were calculated from fold-level test performance values. Because repeated cross-validation folds are not statistically independent, these values are intended to describe empirical fold-level variability rather than independent inferential estimates.*

**Supplementary Table S4. Nested cross-validation sensitivity analysis for LASSO hyperparameter tuning**

| **Feature set** | **Metric** | **Fixed C=10** | **Nested grid search** | **Difference** |
| --- | --- | --- | --- | --- |
| Wearable-only Model | Accuracy | 0.710 | 0.711 | +0.001 |
|  | Recall | 0.288 | 0.277 | -0.011 |
|  | Precision | 0.402 | 0.402 | 0.000 |
|  | F2-score | 0.295 | 0.284 | -0.011 |
| Conventional Assessment Model | Accuracy | 0.743 | 0.745 | +0.002 |
|  | Recall | 0.289 | 0.274 | -0.015 |
|  | Precision | 0.529 | 0.520 | -0.009 |
|  | F2-score | 0.300 | 0.285 | -0.015 |
| Multimodal Fusion Model | Accuracy | 0.773 | 0.778 | +0.005 |
|  | Recall | 0.560 | 0.552 | -0.008 |
|  | Precision | 0.556 | 0.572 | +0.016 |
|  | F2-score | 0.548 | 0.544 | -0.004 |

*Note: Fixed-C results were obtained using the originally specified L1-penalized logistic regression model with C=10. Nested grid-search results were obtained using patient-level nested cross-validation, where C was selected within each outer training fold from C ∈ {0.01, 0.03, 0.1, 0.3, 1, 3, 10, 30, 100} using F2-score as the inner-loop selection criterion. Values represent mean outer validation performance across repeated patient-level validation folds.*

**Supplementary Table S5. Selection frequency of LASSO regularization parameter C in nested cross-validation**

| **Feature set** | **Most frequently selected C** | **C=10 selection rate** | **Interpretation** |
| --- | --- | --- | --- |
| Wearable-only Model | 3 | 31.4% | C=10 was among the frequently selected values |
| Conventional Assessment Model | 1 | 18.2% | C=10 was selected less often but remained within the high-performing range |
| Multimodal Fusion Model | 3 | 21.3% | C=10 achieved the highest mean inner F2 and was within the stable plateau |

**Supplementary Table S6. Standardized LASSO logistic regression coefficients for the multimodal fusion model.**
Coefficients are shown for predictors retained by the L1-penalized logistic regression model after feature standardization. Positive coefficients indicate higher predicted probability of C-SSRS-positive status, whereas negative coefficients indicate lower predicted probability. Coefficients should be interpreted as predictive associations rather than causal effects.

| **Predictor Variable** | **Standardized Coefficient (β)** | **SD (β)** | **Odds Ratio (95% CI)** | **Selection Frequency (%)** |
| --- | --- | --- | --- | --- |
| Act_T_08-10 | -2.329 | 0.735 | 0.12 (0.01–0.23) | 100 |
| first C-SSRS value | 2.197 | 0.648 | 16.19 (4.02–44.91) | 100 |
| sex | -1.643 | 1.377 | 0.31 (0.00–0.59) | 100 |
| Act_Y_22-24 | -1.362 | 0.706 | 0.31 (0.05–0.73) | 99.9 |
| efficiency | 1.151 | 0.481 | 3.75 (1.49–9.18) | 99.9 |
| Act_T_02-04 | 1.031 | 0.453 | 3.34 (1.59–9.20) | 100 |
| Act_Y_06-08 | 0.911 | 0.465 | 2.94 (1.30–7.74) | 99.8 |
| deep_minutes | 0.856 | 0.574 | 2.89 (1.00–8.74) | 96.8 |
| endTime | -0.847 | 0.483 | 0.48 (0.16–1.00) | 93.9 |
| age | -0.736 | 0.522 | 0.55 (0.17–1.35) | 99 |
| Act_Y_12-14 | -0.688 | 0.555 | 0.56 (0.11–1.00) | 98.4 |
| Act_Y_18-20 | 0.54 | 0.555 | 2.23 (0.80–7.31) | 96.8 |
| Act_Y_08-10 | -0.517 | 0.462 | 0.65 (0.20–1.24) | 97.9 |
| Act_Y_20-22 | 0.46 | 0.475 | 1.85 (0.84–5.04) | 96 |
| Act_Y_10-12 | 0.393 | 0.396 | 1.64 (0.76–3.47) | 97.1 |
| light_minutes | -0.39 | 0.458 | 0.74 (0.21–1.00) | 69.3 |
| startTime | 0.389 | 0.382 | 1.62 (1.00–3.70) | 87.1 |
| Act_T_04-06 | 0.38 | 0.417 | 1.62 (0.69–3.82) | 97.6 |
| wake_minutes | -0.241 | 0.716 | 0.95 (0.13–2.00) | 85.5 |
| stay_period | -0.227 | 0.345 | 0.84 (0.37–1.37) | 95.1 |
| Act_T_06-08 | 0.205 | 0.378 | 1.35 (0.65–2.91) | 93.8 |
| rem_minutes | -0.151 | 0.261 | 0.89 (0.43–1.04) | 57.3 |
| Act_T_00-02 | 0.12 | 0.373 | 1.21 (0.51–2.44) | 93.7 |
| Act_Y_16-18 | -0.082 | 0.488 | 1.04 (0.33–2.33) | 94.9 |
| Act_Y_14-16 | -0.047 | 0.364 | 1.03 (0.45–2.03) | 93.7 |
